# The London Ambulance Service

**Published:** 1902-12-20

**Authors:** 


### The Institutional Workshop.

### THE LONDON AMBULANCE SERVICE.

The General Purposes Committee of the London County Council have issued a paper with reference to ambulance provision existing in London and in provincial and continental cities for dealing with cases of accident or sudden illness in the streets or other places. This paper has a prefatory note by Sir William Collins, chairman of the ambulance sub-committee, which is practically as follows :—

1. A consideration of the ambulance service of London naturally falls under the following heads, viz.—

(*a*) A description of the existing services.

(*b*) The question as to whether or not these are adequate.

(*c*) A comparison with the systems in operation in other cities and towns.

2. *The existing services* —The medical officer's report sets out in some detail the present services, from which it will be seen that these are in the hands of the following—(i.) the police; (ii.) the St. John Ambulance Association; (iii) the Bischoffsheim service ; and (iv.) the Volunteer Medical Staff Corps.

The police, who in most cases have the first handling of street accidents, are provided with hand stretchers and wheel litters, and opportunity is afforded to constables for instruction in first aid. For rapid transport only three horsed ambulances exist, maintained by private effort, at Rochester Row, Carter Street, and Stoke Newington police stations, and a charge is made to the public for their use to cover the payment for the horse, driver, and cleansing : these are not kept in constant readiness, but on each occasion, when requisitioned, a horse and driver are supplied from a contractor. The St. John Ambulance Association trains in "first aid" and provides litters and appliances at certain fixed stations, but except at three of these no officer is on duty. The Bischoffsheim service also provides at fire brigade stations and other fixed points wheeled litters and stores, but has no officer on duty. The Volunteer Medical Staff Corps renders valuable ambulance service on rare and special occasions such as public processions. Thus, so far as accidents are concerned, horsed ambulances are practically non-existent in London.

Although much is done by these organisations, there is no uniform adequate and co-ordinate system for dealing with street accidents in London and no central supervising authority in quick touch on the one hand with casualties as they occur and on the other with the hospitals in which they can receive prompt attention. In outlying districts there are naturally fewer facilities than in central ones for dealing with street casualties. The telephone is not utilised. On the other hand, the ambulance arrangements for the conveyance of those certified to be suffering from infectious diseases are already adequately provided by the Metropolitan Asylums Board, and are in striking contrast to this lack of provision for street casualties.

3. *Question of Adequacy.*—In order to ascertain what are the actual means at present in vogue for the conveyance of persons suffering from the results of accidents or sudden illness in the streets or other public places, the sub-committee placed itself in communication with the metropolitan hospitals, some 30 of which receive such cases in London. A record was kept, at the request of the sub-committee, at several of the large London hospitals for a period of four weeks, of the actual mode in which cases of accident and other casualties were brought to the hospitals. Of 994 injured persons arriving at 10 hospitals otherwise than as pedestrians, 642 came in cabs or carts, 302 in different kinds of ambulance, and 50 by other means of conveyance. That is to say, nearly 70 per cent. of the casualties at present conveyed to hospitals by some vehicle or other are taken in cabs or carts and not in ambulances.

From the figures that have been furnished for the year 1899, it would appear that nearly 10,000 street accidents are dealt with by the metropolitan police in the course of the year. In the E Division during the six months ended February, 1902, the police ambulance was used 110 times, the Bischoffsheim ambulances 56 times, and cabs 120 times.

No witness who appeared before the sub-committee considered that the present system of dealing with street casualties in London was adequate. Nearly all advocated the use of more rapid means of transport of the sufferer to the hospital. Horsed or motor ambulances, either in addition to or in substitution of hand-stretchers and litters, were recommended. The use of cabs or carts for casualties where the nature of the injury was either undetermined or such as to be likely to be complicated or augmented by placing the injured person in a cab, was condemned by all. Dr. Perry, the superintendent of Guy's Hospital, said "it is very painful to watch the arrival of accidents at hospitals under the present system." The use of the telephone for prompt summons of rapid means of transport to the injured, and his equally prompt removal to hospital were suggested rather than the elaboration of means of treatment on the spot.

4. *A comparison with the ambulance system in provincial and foreign cities and towns.*—From the medical officer's report it would appear that in some provincial towns and in certain Continental and American cities more modern and efficient systems of ambulance service are at work than is the case in London. Horsed ambulances summoned by telephone are employed at Liverpool, Birkenhead, Manchester, Newcastle, Huddersfield, Bolton, Burnley, Hull, Sheffield, Leeds, and Wolverhampton. In some cases the police, and in others the fire brigade, have the working of the ambulances. The system introduced by Captain Wells, by means of which telephonic messages may be sent from fire-alarm posts, and which has been adapted to the 792 posts in London, has been utilised in Liverpool for the purpose of summoning ambulances in case of street casualties. The mode in which this has been effected will be found in an appendix to Dr. Collie's evidence on page 32. In New York, New Orleans, Paris, and Vienna there are organised systems of town ambulances well worthy of attentive study (see pp. 16–20).

The comparison of London with provincial towns is of course complicated by the fact that in their case there is not the same divided jurisdiction over the police and the fire brigade as there is here. At the present time first-aid is efficiently rendered by the Metropolitan Fire Brigade, but serious cases of burns are handed over to the police and by them removed to hospitals.

Contrast is frequently drawn between the respect which law pays to property in comparison with that which it accords to the person; and while we have in London an elaborate organisation for protecting life and property when threatened with destruction by fire, there is no such adequate and properly organised provision at present available for securing prompt assistance when life and limb are endangered by accident, or when sudden sickness calls for aid in public places.

